# Linkage to care and antiretroviral therapy initiation by testing modality among individuals newly diagnosed with HIV in Tanzania, 2014–2017

**DOI:** 10.1111/tmi.13153

**Published:** 2018-10-24

**Authors:** Christopher T. Rentsch, Alison Wringe, Richard Machemba, Denna Michael, Mark Urassa, Jim Todd, Georges Reniers, Basia Zaba

**Affiliations:** ^1^ Department of Population Health London School of Hygiene & Tropical Medicine London UK; ^2^ The Tazama Project National Institute for Medical Research Mwanza Tanzania; ^3^ MRC/Wits Rural Public Health and Health Transitions Research Unit (Agincourt) School of Public Health Faculty of Health Sciences University of the Witwatersrand Johannesburg South Africa

**Keywords:** HIV, HIV testing, linkage to care, population surveillance, longitudinal studies, sub‐Saharan Africa, VIH, test du VIH, lien vers les soins, surveillance de la population, études longitudinales, Afrique subsaharienne

## Abstract

**Objective:**

To measure linkage to care and antiretroviral therapy (ART) initiation among newly diagnosed individuals with HIV in a rural Tanzanian community.

**Methods:**

We included all new HIV diagnoses of adults made between 2014 and 2017 during community‐ or facility‐based HIV testing and counselling (HTC) in a rural ward in northwest Tanzania. Community‐based HTC included population‐level HIV serological testing (sero‐survey), and facility‐based HTC included a stationary, voluntary HTC clinic (VCT) and an antenatal clinic (ANC) offering provider‐initiated HTC (ANC‐PITC). Cox regression models were used to compare linkage to care rates by testing modality and identify associated factors. Among those in care, we compared initial CD4 cell counts and ART initiation rates by testing modality.

**Results:**

A total of 411 adults were newly diagnosed, of whom 10% (27/265 sero‐survey), 18% (3/14 facility‐based ANC‐PITC) and 53% (68/129 facility‐based VCT) linked to care within 90 days. Individuals diagnosed using facility‐based VCT were seven times (95% CI: 4.5–11.0) more likely to link to care than those diagnosed in the sero‐survey. We found no difference in linkage rates between those diagnosed using facility‐based ANC‐PITC and sero‐survey (*P* = 0.26). Among individuals in care, 63% of those in the sero‐survey had an initial CD4 count >350 cells/mm^3^ *vs*. 29% of those using facility‐based VCT (*P* = 0.02). The proportion who initiated ART within 1 year of linkage to care was similar for both groups (94% sero‐survey *vs*. 85% facility‐based VCT; *P* = 0.16).

**Conclusions:**

Community‐based sero‐surveys are important for earlier diagnosis of HIV‐positive individuals; however, interventions are essential to facilitate linkage to care.

## Abstract (fr)

**Objectif:**

Mesurer le lien entre les soins et l'initiation au traitement antirétroviral (ART) chez les personnes nouvellement diagnostiquées avec le VIH dans une communauté rurale tanzanienne.

**Méthodes:**

Nous avons inclus tous les nouveaux cas d'adultes diagnostiqués avec le VIH entre 2014 et 2017 lors de conseils et de dépistage (CD) du VIH en milieu communautaire ou en établissement dans un quartier rural du nord‐ouest de la Tanzanie. Le CD communautaire comprenait un dépistage sérologique du VIH au niveau de la population (séro‐surveillance) et le CD en établissement comprenait une clinique de conseil et dépistage stationnaire volontaire (CDV) et un CD à l'initiative du prestataire offrant une clinique prénatale (ANC‐CDIS). Les modèles de régression de Cox ont été utilisés pour comparer les taux de ralliement aux soins en testant la modalité pour identifier les facteurs associés. Parmi les personnes en soins, nous avons comparé la numération initiale des CD4 et les taux d'initiation à l'ART par la modalité de test.

**Résultats:**

Un total de 411 adultes ont été nouvellement diagnostiqués, dont 10% (27/265) dans la séro‐ surveillance, 18% (3/14) dans l'ANC‐CDIS basée en établissement et 53% (68/129) dans le CDV en établissement ont été ralliés aux soins dans les 90 jours. Les personnes diagnostiquées à l'aide CDV en établissement étaient sept fois (IC95%: 4,5‐11,0) plus susceptibles d’être ralliées vers les soins que celles diagnostiquées dans la séro‐surveillance. Nous n'avons trouvé aucune différence dans les taux de ralliement entre celles diagnostiquées via l'ANC‐CDIS en établissement et via la séro‐surveillance (*p* = 0,26). Chez les personnes en soins, 63% de celles de la séro‐surveillance avaient une numération initiale des CD4 > 350 cellules/mm^3^ vs 29% de celles utilisant le CDV en établissement (*p* = 0,02). La proportion qui a initié l'ART dans l'année du ralliement aux soins était similaire pour les deux groupes (94% séro‐surveillances contre 85% CDV en établissement; *p* = 0,16).

**Conclusions:**

Les séro‐surveillances communautaires sont importantes pour un diagnostic plus précoce des personnes VIH positives. Cependant, les interventions sont essentielles pour faciliter le lien avec les soins.

## Introduction

HIV testing and counselling (HTC) is the first critical step for subsequent linkage to care and initiating antiretroviral therapy (ART). However, linkage to care following a positive HIV diagnosis remains low in sub‐Saharan Africa. A 2015 meta‐analysis found that linkage to care within 12 months of diagnosis was only 61% (95% confidence interval [CI] 48–72%) among individuals diagnosed using facility‐based, voluntary HTC (VCT) and 55% (95% CI 39–71%) among those diagnosed using facility‐based, provider‐initiated HTC (PITC) 1. Further, linkage to care may vary by throughout the region 2, emphasising the need for locally appropriate interventions to improve linkage to care and subsequent access to ART.

In order to expand access to HIV testing and increase linkage to care and treatment services, WHO recommended community‐based HTC with facilitated linkage to care services (for example, a lay‐counsellor follow up to encourage a clinic visit) in addition to traditional, facility‐based HTC 3. Community‐based HTC includes services that are delivered using mobile and home‐based approaches thus removing structural, logistical and social barriers to HTC 4. While community‐based HTC can increase the number of individuals who knows their HIV status 5, 6, 7, 8, it may also increase the proportion of people living with HIV who knows their status but does not link to HIV care services. A 2015 systematic review found that, only 30% of individuals diagnosed with HIV using mobile and home‐based HTC without facilitated linkage were linked to care within 12 months 1. Major limitations of previous systematic reviews 1, 9 on linkage to care following community‐ and facility‐based HTC are that linkage outcomes were reported without differentiation between newly and previously diagnosed HIV‐positive individuals and the use of both directly observed and self‐reported linkage to care. Individuals who previously tested HIV‐positive and have not yet linked to care are likely to differ from newly identified patients with regard to barriers that may prevent service uptake 10, 11.

Population‐level HIV serological surveys (sero‐surveys) are a unique form of community‐based HTC that include repeated rounds of HIV testing, in which temporary clinics are constructed in numerous locations throughout a community to test all eligible individuals in the population and refer those who test HIV‐positive to register for care at a stationary HIV care and treatment centre (CTC) 12. While diagnoses during a sero‐survey are made relatively closer to participants’ homes, the stationary CTC may be further away, and transportation costs could become a barrier to obtaining care 13. Some sero‐survey systems have offered transportation allowances and volunteer escorts to mitigate such barriers and facilitate linkage to care 14. Although sero‐survey provisions can differ across sites, they are distinct from mobile HTC approaches in four key ways. First, sero‐surveys are often conducted within a surveillance system whereby all eligible residents receive an invitation to participate. Second, in addition to HTC, participants in a sero‐survey are interviewed on a range of topics including family planning, sexual behaviour, HIV testing history and use of ART. Third, participants in a sero‐survey are routinely given a choice whether they want to receive their HIV test results, although sites can differ between using an opt‐in or opt‐out approach. Fourth, repeated rounds of sero‐surveys, with a unique identifier linking all previous test results, allow for the identification of individuals who are newly diagnosed with HIV. The latter two characteristics also serve to distinguish sero‐surveys from sero‐prevalence surveys as they are conducted in Demographic and Health Surveys, which do not disclose results to participants and cannot readily link participation records across multiple rounds. Whether newly diagnosed individuals with HIV during a sero‐survey link to care at different rates than those diagnosed in facility‐based VCT or PITC remains unknown.

In 2015, we introduced a system to link individuals’ sero‐survey records with directly observed HIV testing and care records in a community in northwest Tanzania 15, 16, 17. In this paper, we use the linked data to compare the time from a new HIV‐positive diagnosis to successful linkage to care by testing modality (community‐based HTC provided during sero‐surveys *vs*. facility‐based VCT or PITC provided at stationary clinics). We further explored demographic and spatial characteristics associated with linkage to care. Finally, among those in care, we compared initial CD4 cell count and ART initiation rates by testing modality.

## Methods

### Data sources

**Figure 1:**
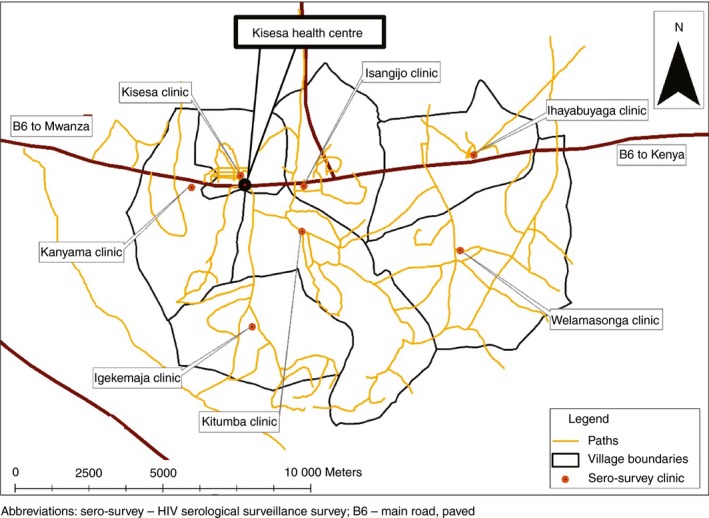
Location of sero‐survey clinics and Kisesa health centre in Kisesa, Tanzania. Sero‐survey – HIV serological surveillance survey; B6 – main road, paved. *Notes*: Kisesa health centre includes the HIV care and treatment centre (CTC), HIV testing and counselling clinic (HTC), and antenatal clinic (ANC); map courtesy of Jocelyn Poppinchalk. [Colour figure can be viewed at wileyonlinelibrary.com]

The Kisesa observational HIV cohort study was established in 1994 and is located in a rural ward in the Magu district of Mwanza region in northwest Tanzania 18. Kisesa ward consists of seven villages with a main road running through large portions of four of the seven villages (Figure 1). The study includes annual or bi‐annual rounds of health and demographic surveillance surveys (HDSS) that cover the entire population of approximately 35 000 residents, and multiple rounds of sero‐surveys, in which adults aged 15 years or older living in the Kisesa HDSS are invited to attend a temporary village‐based clinic for a personal interview and provide blood samples for anonymous HIV testing. The Kisesa sero‐survey system uses an opt‐in approach for offering individuals their HIV test results.

A government‐run health centre is located on the main road within the Kisesa HDSS area, which includes a stationary VCT clinic, an antenatal clinic (ANC) offering PITC (ANC‐PITC) and a CTC. For the stationary VCT and ANC facilities, we developed electronic databases and digitised the paper‐based logbooks using a double‐entry system where two different fieldworkers independently captured each book, and any discrepancy between fields were reconciled in a third cleaning stage. The CTC databases have been fully digitised, and data clerks regularly updated and ran data checks on these data.

### Data linkage

Participants’ records from all sero‐survey rounds are cross‐referenced with their HDSS identifiers as part of the identification process during the survey interview. Records from the three clinics were linked to the HDSS database using point‐of‐contact interactive record linkage (PIRL) 16, 17. Briefly, as individuals arrived any of the three stationary clinics and consented to be in the study, fieldworkers entered the personal and residence details into specialised computer software 15, which used a probabilistic linkage algorithm to search the HDSS database. While searching through potential matches, the fieldworker could view the full list of household members associated with each HDSS record as an additional step to adjudicate true matches. The fieldworker then interacted with the patient to identify which HDSS record(s), if any, was a true match. Among those who reported residence history in the surveillance area, PIRL matched 84% to at least one HDSS record, and this did not differ across the three clinics 17.

### Analytic sample

This analysis included all sero‐survey participants, and HTC and ANC users who received a new HIV‐positive diagnosis between December 2014 and October 2017. Date ranges varied by the source (Table 1). Individuals younger than 15 years of age who received their HIV diagnosis in a clinic were excluded (to be consistent with the 15‐year age limit in the sero‐survey). Individuals were also excluded if their records were not linked with PIRL, had already received a positive HIV diagnostic test, or reported residence outside the HDSS area or were not seen in the 2016/17 HDSS survey (non‐residents). We extracted demographic characteristics including sex, age, rurality of sub‐village (rural, peri‐urban, or urban), whether the sub‐village of residence had a road and geodesic distance between an individual's household and the CTC.

**Table 1:** HIV testing date range and exclusion criteria, by testing modality

	Facility‐based		Community‐based sero‐survey
	ANC‐PITC	VCT	
Minimum HIV+ test date	30/12/2014	15/06/2015	09/09/2015
Maximum HIV+ test date	27/12/2016	03/10/2017	26/02/2016
Number of HIV+ diagnoses	24	159	476
Exclusion criteria			
Previous diagnostic HIV+ test	6 (25.0)	12 (7.6)	204 (42.8)
Non‐resident	1 (4.2)	18 (11.3)	13 (2.7)^a^
Total in analytic sample	17	129	265

HIV, human immunodeficiency virus; ANC, antenatal clinic; PITC, provider‐initiated HIV testing and counselling; VCT, voluntary HIV testing and counselling; sero‐survey, population‐based HIV serological surveillance; HIV+, HIV‐positive. PITC offered through a stationary, antenatal clinic; VCT offered through a stationary, HIV testing and counselling clinic.

a These individuals were residents during the 2015/16 sero‐survey but subsequently moved out of the area.

### Outcomes

The CTC data included all registrations and visits up to November 2017 at Kisesa health centre. The primary outcome was successful linkage to care, defined as the first visit to the CTC including consultation with a clinician within 90 days of diagnosis. Limiting the time frame to 90 days provided a fairer comparison between those who were diagnosed in a health facility and those who were diagnosed in the sero‐survey, given the longer time period between the end of the sero‐survey and available CTC registrations. Among those who linked to care, secondary outcomes were initial CD4 cell count (within one year after linking to care) and ART initiation within 90, 180 or 365 days of linkage to care.

There were other health centres in wards near the Kisesa HDSS surveillance area (all about 5–10 km away from Kisesa health centre via a main road) that offered CTC services during the study period. Of note, this analysis only captured CTC registrations that occurred at Kisesa health centre.

### Statistical analyses

We used chi‐square tests to compare demographic and spatial characteristics between individuals who did and did not link to care. A Cox proportional hazards regression model was used to compare linkage to care rates by testing modality and identify associated factors. Individuals were censored at first CTC visit, death or 90 days after positive HIV diagnosis. We considered all demographic and spatial characteristics and their interaction terms with testing modality for inclusion in an adjusted model. Interaction terms were eliminated from the model using likelihood ratio tests for significance. Remaining terms were assessed for multicollinearity and dropped in a stepwise fashion until there was no further evidence of multicollinearity in the model.

Among those who linked to care, we compared initial CD4 cell counts and the cumulative incidence of ART initiation within 90, 180 and 365 days after having successfully linked to care by testing modality using chi‐square or Fisher's Exact tests. Given the proximity between the CTC and the stationary VCT and ANC clinics (<25 m), we performed sensitivity analysis by excluding individuals who linked to care on the same day as receiving their HIV diagnosis in either of the stationary clinics as all remaining individuals would be required to use transportation to visit the CTC on a subsequent day. This exclusion could be seen as providing a fairer comparison to individuals who obtained community‐based HTC in that those who were diagnosed in a stationary clinic had to return to Kisesa heath centre on a subsequent day. We also performed sensitivity analysis on ART initiation rates by excluding individuals who had a CD4 cell count >500 cells/mm^3^ at CTC registration to mirror treatment guidelines during part of the study period. Statistical analyses were performed using SAS version 9.4 (SAS Institute Inc., Cary, NC, USA).

### Ethics

Ethical approvals were obtained from the Tanzanian National Institute for Medical Research and Lake Zone Institutional Review Board (reference no. NIMR/HQ/R.8c/Vol.II/436 and MR/53/100/450), and the London School of Hygiene and Tropical Medicine (Project ID #8852). Informed written consent was obtained from all participants.

## Results

### Sample characteristics

Between 2014 and 2017, 659 adults received a positive HIV diagnostic test (476 community‐based sero‐surveys, 159 facility‐based VCT, 24 facility‐based ANC‐PITC). After excluding individuals who were previously diagnosed (222/659, 33.7%) and non‐residents (32/659, 4.9%), 411 individuals remained in the analytic sample (265 community‐based sero‐survey, 129 facility‐based VCT, 17 facility‐based ANC‐PITC) (Table 1).

Among the 411 individuals who were newly diagnosed with HIV, 98 (23.8%) linked to care within 90 days of their diagnosis. Of note, only eight individuals in this sample linked to care between 91 and 365 days of diagnosis. By testing modality, linkage to care was higher among those diagnosed using facility‐based VCT (68/129, 52.7%) than those diagnosed using facility‐based ANC‐PITC (3/17, 17.7%) or community‐based sero‐survey (27/265, 10.2%) (*P* < 0.0001) (Table 2). Individuals who resided in Welamasonga (8.9%) or Ihayabuyaga (12.8%) were less likely to link to care than those who resided in Kanyama (33.3%) or Kitumba (35.9%) (*P* = 0.009). There were no significant bivariate associations between linkage to care and sex, age, rurality of sub‐village, whether the sub‐village had a paved road, and distance between household and CTC (all *P* > 0.09). However, after excluding individuals who linked to care on the same day as receiving their HIV diagnosis, there was a statistically significant association between linkage to care and distance between household and CTC (16.0% for <1 km, 21.9% for 1–1.9 km, and 20.7% for 2–4.9 km; *P* = 0.03).

**Table 2:** Characteristics of individuals who received their first positive HIV diagnosis between 2015 and 2017 in Kisesa, Tanzania, by whether they subsequent linked to care

Characteristic	Linked to care	Did not link to care	*P*‐value
	(*n* = 98)	(*n* = 313)	
Testing modality			
Facility‐based VCT	68 (52.7)	61 (47.3)	<0.0001
Facility‐based ANC‐ PITC	3 (17.7)	14 (82.3)	
Community‐based sero‐survey	27 (10.2)	238 (89.8)	
Sex			
Male	40 (28.8)	99 (71.2)	0.0934
Female	58 (21.3)	214 (78.7)	
Age, years			
15–29	31 (25.8)	89 (74.2)	0.9237
30–39	35 (23.8)	112 (76.2)	
40–49	18 (22.5)	62 (77.5)	
50+	14 (21.9)	50 (78.1)	
Village			
Igekemaja	12 (26.1)	34 (73.9)	0.0094
Ihayabuyaga	5 (12.8)	34 (87.2)	
Isangijo	8 (19.5)	33 (80.5)	
Kanyama	20 (33.3)	40 (66.7)	
Kisesa	26 (22.4)	90 (77.6)	
Kitumba	23 (35.9)	41 (64.1)	
Welamasonga	4 (8.9)	41 (91.1)	
Rurality of sub‐village			
Urban	24 (22.0)	85 (78.0)	0.1496
Peri‐urban	30 (31.3)	162 (78.6)	
Rural	44 (21.4)	66 (68.8)	
Sub‐village has paved road			
Yes	44 (25.7)	127 (74.3)	0.4487
No	54 (22.5)	186 (77.5)	
Distance from household to CTC, km			
<1	19 (23.2)	63 (76.8)	0.1214
1–1.9	32 (29.9)	75 (70.1)	
2–4.9	24 (27.0)	65 (73.0)	
5–11	23 (17.3)	110 (82.7)	

VCT, voluntary HIV testing and counselling; ANC, antenatal clinic; PITC, provider‐initiated HIV testing and counselling; sero‐survey – population‐based HIV serological surveillance; CTC, HIV care and treatment centre. All statistics are given in *n* (%); differences assessed using chi‐square tests.

### Associations with linkage to care

Median time from diagnoses to linkage to care was 20 days (interquartile range [IQR] 4–47 days) among those diagnosed in the community‐based sero‐survey and 1 day (IQR 1–14 days) among those diagnosed using facility‐based VCT. All three individuals diagnosed using facility‐based ANC‐PITC who linked to care did so on the same day as diagnosis. In an unadjusted model, individuals diagnosed using facility‐based VCT were seven times more likely to link to care than those diagnosed in the community‐based sero‐survey (hazard ratio [HR] 7.01, 95% CI 4.47–10.97) (Table 3). There was no statistical evidence that individuals diagnosed using facility‐based ANC‐PITC linked to care at higher rates than those diagnosed in the community‐based sero‐survey (HR 1.90, 95% CI 0.58–6.27).

**Table 3:** Associations with linkage to care among individuals receiving their first HIV+ diagnosis in a population‐based HIV serological survey or health facility in Kisesa, Tanzania between 2014 and 2017, *n* = 411

Covariate	cHR (95% CI)	aHR (95% CI)
Testing modality		
Facility‐based VCT	7.01 (4.47–10.97)***	6.95 (4.39–11.00)***
Facility‐based ANC‐PITC	1.90 (0.58–6.27)	2.00 (0.59–6.75)
Community‐based sero‐survey	1	1
Sex		
Male	1.40 (0.93–2.09)	1.44 (0.93–2.23)*
Female	1	1
Age, years		
15–29	1.19 (0.63–2.24)	0.97 (0.50–1.86)
30–39	1.08 (0.58–2.01)	1.12 (0.59–2.11)
40–49	1.01 (0.50–2.02)	1.10 (0.54–2.25)
50+	1	1
Village		
Igekemaja	3.15 (1.02–9.78)*	
Ihayabuyaga	1.44 (0.39–5.35)	
Isangijo	2.21 (0.67–7.33)	
Kanyama	4.26 (1.46–12.47)**	–
Kisesa	2.59 (0.90–7.41)	
Kitumba	4.74 (1.64–13.70)**	
Welamasonga	1	
Rurality of sub‐village		
Urban	1.01 (0.61–1.66)	0.46 (0.16–1.33)
Peri‐urban	1.57 (0.99–2.50)	0.91 (0.42–1.95)
Rural	1	1
Sub‐village has paved road		
Yes	1.12 (0.75–1.67)	1.10 (0.59–2.07)
No	1	1
Distance from household to CTC, km		
<1	1.36 (0.74–2.49)	2.22 (0.76–6.45)
1–1.9	1.85 (1.08–3.16)*	1.86 (0.80–4.34)
2–4.9	1.62 (0.91–2.86)	1.40 (0.76–2.59)
5–11	1	1

HIV, human immunodeficiency virus; HIV+, HIV‐positive; cHR, crude unadjusted hazard ratio; aHR, adjusted hazard ratio; CI, confidence interval; VCT, voluntary HIV testing and counselling; ANC, antenatal clinic; PITC, provider‐initiated HIV testing and counselling; sero‐survey, population‐based HIV serological surveillance; CTC, HIV care and treatment centre; km, kilometres.

*P < 0.05; **P < 0.01; ***P < 0.0001.

**Figure 2:**
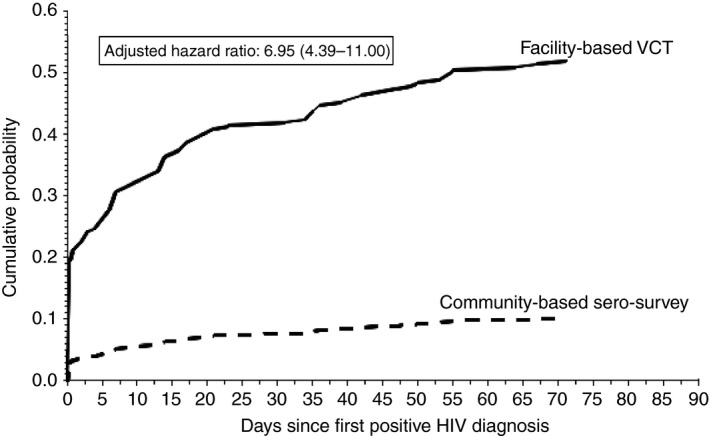
Adjusted cumulative probability of registration for HIV care after first positive HIV diagnosis by testing modality in Kisesa, Tanzania between 2014 and 2017, *n* = 411. VCT – voluntary counselling and testing; facility‐based provider‐initiated counselling and testing had too few individuals for curve to be drawn; allowed for 90 days of follow‐up, no event >71 days; adjusted for sex, age, rurality of sub‐village (urban, peri‐urban, rural), whether the sub‐village had access to a paved road, and distance between household and Kisesa health centre.

The final adjusted model included sex, age, rurality of sub‐village (urban, peri‐urban, rural), whether the sub‐village had access to a paved road and distance between household and CTC. The associations between linkage to care and testing modality remained after adjustment (HR 6.95, 95% CI 4.39–11.00 facility‐based VCT; HR 2.00, 95% CI 0.59–6.75 facility‐based ANC‐PITC) (Figure 2). No other significant associations were found after adjustment.

### Initial CD4 count and ART initiation

Among individuals who linked to care, the proportion of individuals whose initial CD4 cell count was >500 cells/mm^3^ was higher among those diagnosed in the community‐based sero‐survey (42%) than facility‐based VCT (16%) (*P* = 0.05). None of the three individuals diagnosed using facility‐based ANC‐PITC had a CD4 laboratory result on record.

Among the 68 individuals diagnosed using facility‐based VCT and linked to care, 55 of 68 (80.9%) initiated ART within 90 days after linking to care, 59/68 (86.8%) initiated ART within 180 days after linking to care, and 64 of 68 (94.1%) initiated ART within 365 days linking to care. Among the 27 individuals diagnosed in the community‐based sero‐survey and linked to care, 17 of 27 (63.0%) initiated ART within 90 days after linking to care, 21 of 27 (77.8%) initiated ART within 180 days after linking to care and 23 of 27 (85.2%) initiated ART within 365 days after linking to care. All three individuals who were diagnosed using facility‐based ANC‐PITC and linked to care initiated ART on their first visit to the CTC. At each time window, there was no statistically significant difference by testing modality of the proportion of individuals who, having linked to care, initiated ART (all *P* > 0.11). The proportion of individuals initiating ART increased further, yet conclusions remained the same, when restricting to those whose initial CD4 cell count was <500 cells/mm^3^, which was the national guideline for when to initiate treatment for part of the study period.

### Sensitivity analysis

Of the 71 individuals diagnosed using facility‐based HTC (68 VCT and 3 ANC‐PITC), 38 (54%) linked to care on the same day they received their HIV diagnosis (the ANC, VCT and CTC are located within the same health centre). After excluding these individuals from the adjusted model (which included all three from facility‐based ANC‐PITC), the association between testing modality and linkage to care remained, although attenuated (HR 3.89, 95% CI 2.30–6.58 facility‐based VCT vs. community‐based sero‐survey). In this restricted model, there was a clear stepped increase in the likelihood of linkage to care by proximity between household and the CTC. Compared to individuals whose households were ≥5 km away from the CTC, those who lived closer to the CTC were significantly more likely to link to care (HR 4.67, 95% CI 1.16–18.76 for <1 km; HR 4.69, 95% CI 1.51–14.56 for 1–1.9 km; HR 2.66, 95% CI 1.17–6.06 for 2–4.9 km).

## Discussion

Linkage to care within 90 days was low (24%) among newly diagnosed adults with HIV between 2014 and 2017 in this rural Tanzanian population. However, individuals who received their first HIV diagnosis using facility‐based VCT had seven‐fold greater linkage to care than individuals diagnosed using community‐based sero‐surveys. Among individuals who linked to HIV care services, individuals diagnosed in the community‐based sero‐survey had proportionately higher initial CD4 cell counts >500 cells/mm^3^ than those diagnosed using facility‐based VCT. However, ART initiation rates among those who linked to care were similar irrespective to HIV testing modality. While sero‐surveys are important for expanding testing coverage and identifying more recent infections, our findings highlight the need for additional interventions to help individuals who are diagnosed with HIV in a community‐based sero‐survey round link to care and treatment.

The low level of linkage to care in our sample is concerning, particularly among those diagnosed during the sero‐survey, but is comparable to those documented in South Africa 19 and Zambia 20. Our finding of higher uptake of HIV care services among individuals diagnosed using facility‐based VCT than those diagnosed using community‐based HTC is corroborated by a 2015 meta‐analysis of studies reporting rates of linkage to care throughout sub‐Saharan Africa. Pooling data from 31 studies, community‐based HTC achieved approximately 30% linkage, facility‐based PITC (overall and not restricted to ANC‐PITC) achieved 55% linkage and facility‐based VCT achieved 61% linkage 1, *vs*. our findings of 10%, 18% and 53% respectively. The overall higher uptake of linkage to care in the meta‐analysis could be due to a number of factors. First, the studies in the systematic review did not differentiate newly diagnosed individuals from those who had previously obtained a positive HIV test result. It is plausible that individuals who have received multiple positive test results may be more likely to seek HIV care services than those diagnosed for the first time. Second, the method of ascertainment of linkage to care included participant self‐reports, which may be affected by social desirability bias 21. Third, the meta‐analysis included studies that followed individuals up to 1 year to identify successful linkage compared to 90 days in our study. However, very few individuals in our sample linked to care between 91 and 365 days. Fourth, our sample for facility‐based PITC was restricted to ANC users, whereas users of other facilities offering PITC, such as outpatient clinics, may include sicker individuals. Finally, uptake of HIV care services in Kisesa has consistently lagged behind other eastern and southern African communities, likely due to community‐level stigma and other social and structural barriers 2, 8, 14, 22. Notably, the systematic review found that community‐based HTC accompanied by facilitated linkage to care by trained lay counsellors or health workers achieved 95% linkage within 12 months 1. Therefore, future sero‐surveys should explore including facilitated linkage to care among those diagnosed with HIV as a way to improve linkage to care.

We found that individuals who resided in villages in the eastern region of the study area (i.e. Ihayabuyaga and Welamasonga) were less likely to link to care than those in the other villages. In addition, linkage to care rates were higher in Kitumba and Kanyama than in Kisesa village in which the CTC is located. Given Kisesa village's proximity with the CTC, we hypothesise that individuals who lived in the immediate area surrounding the clinics may have been more likely to travel to obtain HIV care outside of the surveillance area following a diagnosis made within Kisesa, which would have resulted in the attenuated effect. There is some evidence for this in a previous study of ours that showed nearly half of all clinic attendees in the CTC between 2015 and 2017 were non‐residents 17, which underscores the importance for future trials and observational studies to include tracing of diagnosed individuals to capture care received outside the immediate area, where possible. Although the proximity of each village to the CTC is likely to be a key factor driving the differential linkage to care rates by village, other factors through which village of residence may be acting include access to CTC facilities outside the study area and social factors like community‐level stigma.

Previous studies have highlighted the success of community‐based HTC to identify asymptomatic HIV‐positive individuals at relatively higher CD4 counts compared with facility‐based HTC 1, 9, 23. Our findings are consistent with these previous studies in that nearly half of individuals diagnosed in the community‐based sero‐survey had CD4 counts >500 cells/mm^3^ at care initiation compared to only 16% of those diagnosed using facility‐based VCT. Of note, 74% of all newly diagnosed individuals in Kisesa who linked to care between 2014 and 2017 had CD4 < 500 cells/mm^3^ when they initiated care. National treatment guidelines in Tanzania were to initiate ART in individuals with CD4 < 500 cells/mm^3^ in 2015 24, which expanded to all diagnosed individuals in 2017 25 to match current WHO guidelines 26. Therefore, most individuals in our sample were eligible for treatment when they linked to care, which corresponds with the high level of ART initiation we observed among those who did.

We conducted a sensitivity analysis on our regression model to exclude individuals who were diagnosed and linked to care in the same day. In this restricted model, we still found a strong, albeit attenuated, association between testing modality and linkage to care. We also found the distance between an individual's household and the CTC played a role in the likelihood of successfully linking to care after restricting to those who would have needed to travel back to the CTC on a subsequent day to achieve linkage to care. These findings further underscore the importance of providing equal access to HIV care and treatment services irrespective of the distance from the nearest stationary CTC, including transportation refunds. By the end of 2017, three village‐based health posts located within the Kisesa HDSS surveillance area were offering ART directly to attendees in addition to the CTC at Kisesa health centre. We are currently assessing how best to link clinic records from these less‐frequented health posts into the linked data infrastructure.

Our study had limitations. First, our analysis did not capture linkage to care that occurred outside the study area so the proportion who linked to care is likely to be underestimated. If the decision to obtain care outside of Kisesa ward was related to modality of HIV testing or any spatial characteristic (as may have been the case for Kisesa village), the results in this paper may be subjected to bias. We used all available data to control for such bias, including limiting the analytic sample to those who were resident in the study area as of 2017. Second, we lacked sufficient power because of the relatively small sample size, particularly among those diagnosed using facility‐based ANC‐PITC, which resulted in large standard errors of regression estimates. A larger number of individuals who had already been diagnosed with HIV in the facility‐based sample may have also allowed for a separate analysis among these individuals to identify their likelihood of linkage to care after multiple diagnoses.

## Conclusion

We measured linkage to care following a population‐level HIV serological surveillance round, which is a form of community‐based HTC not previously included in systematic reviews on the topic. This analysis was made possible and strengthened by the novel linked data infrastructure available in the Kisesa observational HIV cohort study, which includes directly observed data for HIV testing (both community‐ and facility‐based), diagnoses, care, and treatment. We found that while overall linkage to care was low among newly diagnosed adults in this rural Tanzanian community, those diagnosed using facility‐based VCT had higher uptake of HIV care services than those diagnosed using facility‐based ANC‐PITC or in the community‐based sero‐survey. However, once individuals were in care, there was no evidence of any further delays to ART initiation by testing modality. Community‐based HTC is important for earlier diagnosis of HIV‐positive individuals; however, these efforts should include interventions to link individuals newly diagnosed with HIV into care and provide stationary care and treatment services in all locations offering HTC.

## Data availability

Due to ethical clearances, the data sets used and analysed during the current study are not publicly available. The linkage algorithm requires personally identifiable information, which our ethics certificate restricts from sharing. However, applications to access portions of the data that can be anonymised for collaborative analysis are encouraged and can be made by contacting the project coordinator for the Kisesa HIV serological surveillance, Mark Urassa (urassamark@yahoo.co.uk), or by contacting the ALPHA Network team (alpha@lshtm.ac.uk; http://alpha.lshtm.ac.uk/).
